# The mouse–canine chimeric anti-dog podoplanin antibody P38B exerts antitumor activity in mouse xenograft models

**DOI:** 10.1016/j.bbrep.2018.11.005

**Published:** 2018-11-24

**Authors:** Yukinari Kato, Tomokazu Ohishi, Manabu Kawada, Naoya Maekawa, Satoru Konnai, Shunsuke Itai, Shinji Yamada, Mika K. Kaneko

**Affiliations:** a Department of Antibody Drug Development, Tohoku University Graduate School of Medicine, 2-1 Seiryo-machi, Aoba-ku, Sendai, Miyagi 980-8575, Japan; b New Industry Creation Hatchery Center, Tohoku University, 2-1 Seiryo-machi, Aoba-ku, Sendai, Miyagi 980-8575, Japan; c Institute of Microbial Chemistry (BIKAKEN), Numazu, Microbial Chemistry Research Foundation, 18-24 Miyamoto, Numazu-shi, Shizuoka 410-0301, Japan; d Department of Advanced Pharmaceutics, Faculty of Veterinary Medicine, Hokkaido University, Kita 18, Nishi 9, Kita-ku, Sapporo 060-0818, Japan; e Laboratory of Infectious Diseases, Department of Disease Control, Faculty of Veterinary Medicine, Hokkaido University, Kita 18, Nishi 9, Kita-ku, Sapporo 060-0818, Japan

**Keywords:** ADCC, antibody-dependent cellular cytotoxicity, CDC, complement-dependent cytotoxicity, CHO, Chinese hamster ovary, CLEC-2, C-type lectin-like receptor 2, dPDPN, dog podoplanin, mAb, monoclonal antibody, PBS, phosphate-buffered saline, PDPN, podoplanin, SCC, squamous cell carcinomas, Mouse-canine chimeric antibody, Dog podoplanin, dPDPN, Monoclonal antibody

## Abstract

Podoplanin (PDPN) is a type I transmembrane heavily glycosylated sialoglycoprotein that is expressed in normal tissues such as pulmonary type I alveolar cells, renal podocytes, and lymphatic endothelial cells. PDPN overexpression in cancerous tissue is associated with hematogenous metastasis through interactions with the C-type lectin-like receptor 2 (CLEC-2). Previously, we have reported the development of a mouse monoclonal antibody (mAb), PMab-38 (IgG_1_, kappa) against dog PDPN (dPDPN). PMab-38 was found to strongly react with canine squamous cell carcinomas (SCCs) and melanomas; however, it showed no reaction with lymphatic endothelial cells. Recently, we have developed and produced the mouse–canine mAb of subclass B, P38B that showed antibody-dependent cellular cytotoxicity (ADCC) and complement-dependent cytotoxicity against Chinese hamster ovary (CHO)/dPDPN cells. In the present study, we investigated the antitumor activity using mouse xenograft model. To induce ADCC activity by P38B, canine mononuclear cells were injected surrounding the tumors in a xenograft model. It was demonstrated that P38B exerted antitumor activity against the mouse xenograft model using CHO/dPDPN. These results suggest that P38B is useful for antibody therapy against dPDPN-expressing canine SCCs and melanomas.

## Highlights

• Dog PDPN is expressed in canine squamous cell carcinomas and melanomas.
• A mouse-canine mAb of canine subclass B, P38B against dog PDPN was produced.
• P38B exerted antitumor activities via ADCC and CDC.
• P38B could be useful for antibody therapy against dPDPN-expressing canine tumors.

## 1 Introduction

Podoplanin (PDPN) is expressed in normal tissue, including pulmonary type I alveolar cells, renal podocytes, chondrocytes, myofibroblasts, mesothelial cells, and lymphatic endothelial cells [1]. PDPN overexpression is also observed in different types of tumors, including mesotheliomas [2], [3], squamous cell carcinomas (SCC) [4], testicular tumors [5], and glioblastomas [6]. Recent clinical studies have provided evidence for the association between increased PDPN expression and poor disease prognosis [7]. This observation indicated that the establishment of anti-PDPN monoclonal antibodies (mAbs) is critical for developing novel therapeutic strategies against cancer development and metastatic progression [8].

Dog PDPN (dPDPN) was previously reported in the literature as gp40 [9]. We have recently developed two mAbs PMab-38 (mouse IgG_1_, kappa) [10] and PMab-48 (mouse IgG_1_, kappa) [11]. It was shown that PMab-38 recognized dPDPN of renal epithelial cells weakly but showed no reaction with lymphatic endothelial cells [10]. Conversely, PMab-48 reacted with renal epithelial cells as well as with lymphatic endothelial cells [11]. Tyr67 and Glu68 of dPDPN were determined as the critical epitopes of PMab-38 [12], whereas Asp29, Asp30, Ile31, Ile32, and Pro33 of dPDPN were essential for the recognition of PMab-48 [13]. Immunohistochemistry demonstrated that PMab-38 reacted with 83% of canine SCCs (15/18 cases) [14] and 90% of melanomas (9/10 cases) [15]. Recently, we have developed a mouse–canine chimeric antibody (P38B) that was derived from PMab-38 [16]. P38B demonstrated antibody-dependent cellular cytotoxicity (ADCC) and complement-dependent cytotoxicity (CDC) against CHO/dPDPN cells, indicating that P38B is applicable for antibody-based therapy for canine cancers. In the present study, we investigated antitumor activity against mouse xenograft models using CHO/dPDPN and CHO-K1.

## 2 Materials and methods

### 2.1 Cell lines

Chinese hamster ovary (CHO)-K1 cell-line was obtained from the American Type Culture Collection (ATCC; Manassas, VA). In our previous studies, we have inserted dPDPN with a N-terminal PA tag and a C-terminal RAP tag-MAP tag (PA-dPDPN-RAP-MAP) in a pCAG-Ble vector (FUJIFILM Wako Pure Chemical Corporation, Osaka, Japan) [10]. The PA tag [17], RAP tag [18], and MAP tag [19] comprises 12 amino acids each—GVAMPGAEDDVV, DMVNPGLEDRIE, and GDGMVPPGIEDK, respectively. CHO-K1 cells were transfected with pCAG-Ble/PA-dPDPN-RAP-MAP using Gene Pulser Xcell electroporation system (Bio-Rad Laboratories Inc., Berkeley, CA) resulting in the cell-line CHO/dPDPN. CHO-K1 and CHO/dPDPN were cultured in RPMI 1640 medium (Nacalai Tesque, Inc., Kyoto, Japan) supplemented with 10% heat-inactivated fetal bovine serum (Thermo Fisher Scientific Inc., Waltham, MA), 100 units/mL of penicillin, 100 μg/mL of streptomycin, and 25 μg/mL of amphotericin B (Nacalai Tesque, Inc.) at 37 °C in a humidified atmosphere of 5% CO_2_ and 95% air.

### 2.2 Antibodies

The mouse anti-dPDPN mAb PMab-38 was developed as previously described [10]. To generate the mouse–canine (subclass B) chimeric antibody P38B, the appropriate V_H_ and V_L_ cDNAs of mouse PMab-38 and the C_H_ and C_L_ of canine IgG subclass B were subcloned into pCAG-Ble and pCAG-Neo vectors (FUJIFILM Wako Pure Chemical Corporation), respectively [16]. Expression vectors were transfected into ExpiCHO-S cells using the ExpiFectamine CHO Transfection kit (Thermo Fisher Scientific Inc.) to express P38B antibody, which was further purified using Protein G-Sepharose (GE Healthcare Bio-Sciences, Pittsburgh, PA, USA).

### 2.3 Antitumor activity of P38B

Female BALB/c nude mice (5-week-old) were purchased from Charles River (Kanagawa, Japan) and used in experiments when they were 7 weeks old. CHO/dPDPN and CHO-K1 cells (0.3 mL of 1.33 × 10^8^ /mL in RPMI) were mixed with 0.5 mL of BD Matrigel Matrix Growth Factor Reduced (BD Biosciences, San Jose, CA, USA). A 100-μL suspension (containing 5 × 10^6^ cells) was injected subcutaneously into the left flanks of nude mice. After day 1, 100 μg of P38B or dog IgG (Jackson ImmunoResearch Inc., PA, USA) in 100 μL PBS were injected into the peritoneal cavity of each mouse. Additional antibodies were injected on day 8 and day 15. Canine mononuclear cells (5 x 10^5^ cells), which were obtained from Hokkaido University, were injected surrounding the tumors on day 1, day 8, and day 15. The tumor diameter and tumor volume were determined as previously described [20]. The mice were euthanized 17 days after cell implantation. All data were expressed as the mean ± SEM. Statistical analysis was performed using the Tukey–Kramer test. *P* < 0.05 was considered statistically significant.

## 3 Results and discussion

We have previously reported the development of the mouse mAb, PMab-38 against dPDPN [10]. The strong reaction of PMab-38 specifically with canine SCCs [15] and melanomas [16] indicated that PMab-38 possesses cancer-specificity. We have previously established cancer-specific mAbs against human PDPN, such as LpMab-2 [8] and LpMab-23 [21]. These cancer-specific mAbs may prove advantageous for targeting cancer cells without adverse events.

To date, few studies have been performed on the subclasses (A, B, C, D) of canine IgGs [22], [23]. However, it was demonstrated that the canine subclasses A and D appear to be effector-function negative, whereas subclasses B and C bind to canine Fc gamma receptors and are positive for ADCC similar to human IgG_1_ and IgG_3_, respectively [22]. Furthermore, subclasses B and C can induce CDC. An anti-canine CD20 mAb (1E4) was established to treat canine B-cell lymphoma and produced mouse–canine chimeric antibodies [23]. It was also shown that 1E4-cIgGB (subclass B) and 1E4-cIgGC (subclass C) led to significant depletion of B-cell levels in healthy beagle dogs. Moreover, we produced P38B, a mouse–canine chimeric antibody of canine subclass B, from PMab-38 [16]. In that study, we clearly demonstrated that P38B possesses ADCC and CDC against CHO/dPDPN cells.

**Fig. 1:**
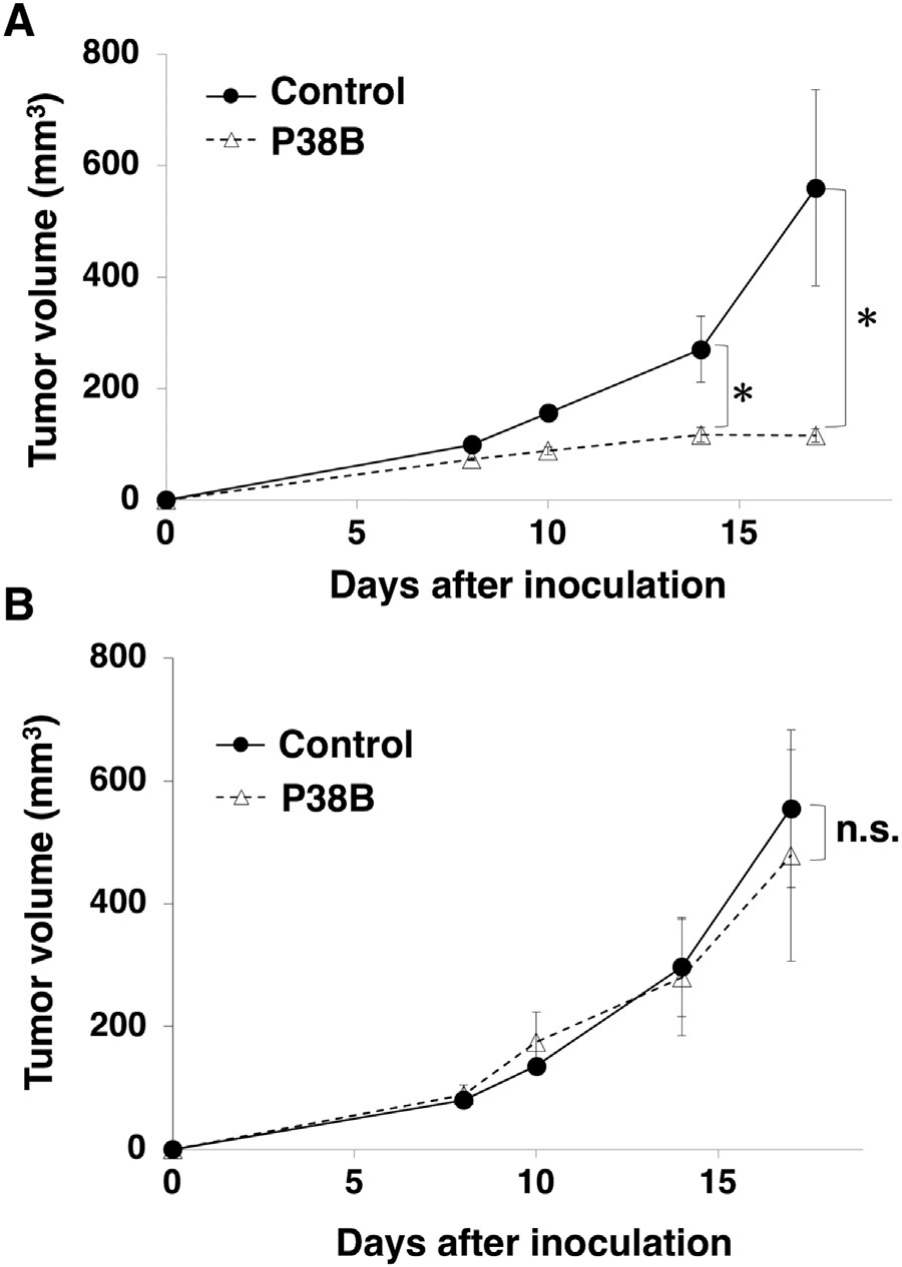
**Antitumor activity of P38B against CHO/dPDPN and CHO-K1. (A)** Tumor volume of CHO/dPDPN xenografts. CHO/ dPDPN cells were injected subcutaneously into female nude mice. The indicated antibodies (100 μg/day; 5 mg/kg) were administered intraperitoneally on days 1, 8, and 15 after cell inoculation. The tumor volume was measured at the indicated time points. The values are presented as mean ± SEM. **(B)** CHO-K1 cells were injected subcutaneously into female nude mice. The indicated antibodies (100 μg/day; 5 mg/kg) were administered intraperitoneally, and canine mononuclear cells were injected around the tumors on day 1, day 8, and day 15. The tumor volume was measured at the indicated time points. The values are presented as mean ± SEM. An asterisk indicates statistical significance (* *P* < 0.05, Tukey-Kramer's test). n.s.: not significant.

To study the antitumor activity of P38B on cell growth in vivo, CHO/dPDPN cells or CHO-K1 cells were subcutaneously implanted into the flanks of nude mice. P38B and control dog IgG were injected three times (on day 1, 8, and 15 after cell injections) into the peritoneal cavity of mice. Canine mononuclear cells were injected three times (on day 1, 8, and 15) surrounding the xenograft. Tumor formation was observed in mice from the control and P38B-treated groups in CHO/dPDPN xenograft models (Fig. 1A) and CHO-K1 xenograft models (Fig. 1B). However, P38B significantly reduced tumor development of CHO/dPDPN xenograft compared with control dog IgG on day 14 and day 17 (Fig. 1A). Conversely, P38B did not reduce tumor development of CHO-K1 xenograft (Fig. 1B). This observation indicated that P38B is specific against dPDPN.

**Fig. 2:**
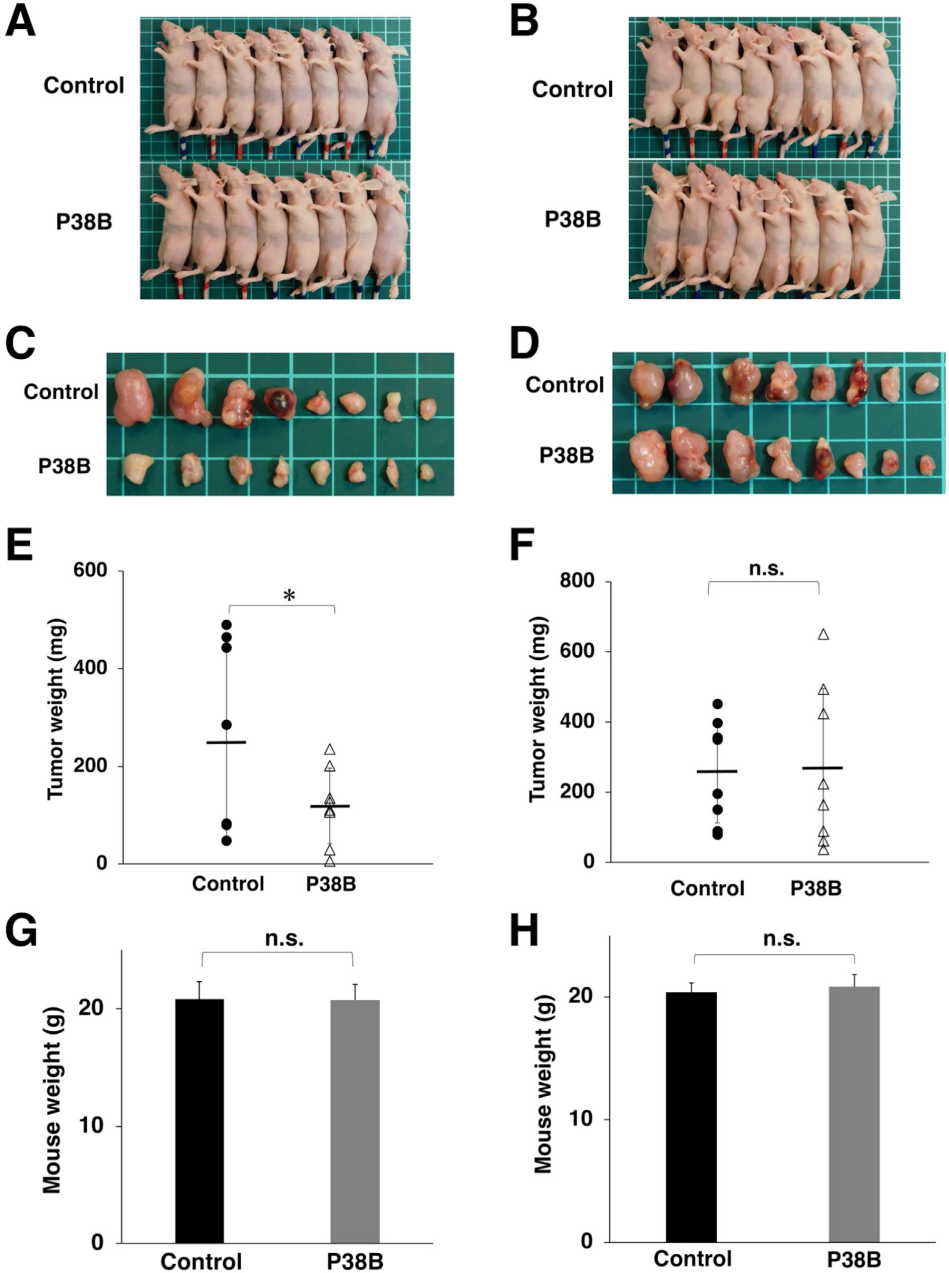
**Evaluation of antitumor activity of P38B against CHO/dPDPN and CHO-K1. (A)** CHO/dPDPN xenograft mice models on day 17. **(B)** CHO-K1 xenograft mice models on day 17. **(C)** Resected tumors of CHO/dPDPN xenografts. **(D)** Resected tumors of CHO-K1 xenografts. **(E)** Tumor weight of CHO/dPDPN xenografts (day 17). **(F)** Tumor weight of CHO-K1 xenografts (day 17). (**G**) Body weight of CHO/dPDPN xenografts (day 17). (**H**) Body weight of CHO-K1 xenografts (day 17). The values are presented as mean ± SEM. An asterisk indicates statistical significance (* *P* < 0.05, Tukey–Kramer's test). n.s.: not significant.

CHO/dPDPN and CHO-K1 xenograft mice models on day 17 are shown in Fig. 2A and B, respectively. The resected tumors of CHO/dPDPN and CHO-K1 xenografts are depicted in Fig. 2C and D, respectively. The tumor weight of mice in P38B-treated was significantly lower than in the control dog IgG group in CHO/dPDPN xenograft models (Fig. 2E) although there was no difference in CHO-K1 xenograft models (Fig. 2F). However, body weight was not significantly different among the two groups in the CHO/dPDPN xenograft models (Fig. 2G) and the CHO-K1 xenograft models (Fig. 2H).

In conclusion, P38B is applicable for antibody therapy against canine cancers expressing dPDPN. Further studies on antitumor activities against endogenous dPDPN-expressing tumors are necessary to obtain a more detailed understanding of antibody therapy against canine cancers.
